# Nephronectin as a Matrix Effector in Cancer

**DOI:** 10.3390/cancers13050959

**Published:** 2021-02-25

**Authors:** Synnøve Norvoll Magnussen, Jimita Toraskar, Elin Hadler-Olsen, Tonje S. Steigedal, Gunbjørg Svineng

**Affiliations:** 1 Department of Medical Biology, Faculty of Health Sciences, UiT—The Arctic University of Norway, 9037 Tromsø, Norway; elin.hadlerolsen@tffk.no (E.H.-O.); gunbjorg.svineng@uit.no (G.S.); 2 Department of Clinical and Molecular Medicine, Faculty of Medicine and Health Sciences, Norwegian University of Science and Technology (NTNU), 7491 Trondheim, Norway; toraskarjimita@gmail.com (J.T.); tonje.s.steigedal@ntnu.no (T.S.S.); 3 Cancer Clinic, St. Olavs Hospital HF, 7006 Trondheim, Norway; 4 The Public Dental Health Service Competence Center of Northern Norway, 9271 Tromsø, Norway

**Keywords:** nephronectin, cancer, breast cancer, extracellular matrix, metastasis, cancer progression

## Abstract

**Simple Summary:**

The extracellular matrix provides an important scaffold for cells and tissues of multicellular organisms. The scaffold not only provides a secure anchorage point, but also functions as a reservoir for signalling molecules, sequestered and released when necessary. A dysregulated extracellular matrix may therefore modulate cellular behaviour, as seen during cancer progression. The extracellular matrix protein nephronectin was discovered two decades ago and found to regulate important embryonic developmental processes. Loss of either nephronectin or its receptor, integrin α8β1, leads to underdeveloped kidneys. Recent findings show that nephronectin is also dysregulated in breast cancer and plays a role in promoting metastasis. To enable therapeutic intervention, it is important to fully understand the role of nephronectin and its receptors in cancer progression. In this review, we summarise the literature on nephronectin, analyse the structure and domain-related functions of nephronectin and link these functions to potential roles in cancer progression.

**Abstract:**

The extracellular matrix protein nephronectin plays an important regulatory role during embryonic development, controlling renal organogenesis through integrin α8β1 association. Nephronectin has three main domains: five N-terminal epidermal growth factor-like domains, a linker region harbouring two integrin-binding motifs (RGD and LFEIFEIER), and a C-terminal MAM domain. In this review, we look into the domain-related functions of nephronectin, and tissue distribution and expression. During the last two decades it has become evident that nephronectin also plays a role during cancer progression and in particular metastasis. Nephronectin is overexpressed in both human and mouse breast cancer compared to normal breast tissue where the protein is absent. Cancer cells expressing elevated levels of nephronectin acquire increased ability to colonise distant organs. In particular, the enhancer-motif (LFEIFEIER) which is specific to the integrin α8β1 association induces viability via p38 MAPK and plays a role in colonization. Integrins have long been desired as therapeutic targets, where low efficiency and receptor redundancy have been major issues. Based on the summarised publications, the enhancer-motif of nephronectin could present a novel therapeutic target.

## 1. The Extracellular Matrix as a Regulator of Tissue Homeostasis

The extracellular matrix (ECM) is a complex and dynamic non-cellular component of tissues performing many critical functions. The ECM maintains tissue integrity by providing a scaffold while also absorbing mechanical tension. Through ECM anchoring, cells are provided with cues that either maintain tissue homeostasis or induce cellular changes, such as those seen during development and wound healing. The ECM is composed of more than 300 different proteins that can be broadly divided into collagens, proteoglycans, glycoproteins and other ECM associated proteins [1]. These include ECM-sequestered growth factors, cytokines and chemokines. The ECM hence functions as a reservoir that provides cells with signalling cues for growth, survival, proliferation, polarity, migration and differentiation [1,2,3,4]. Alterations in the composition of the ECM may therefore impact greatly on cellular behaviour as seen during cancer progression [5].

Cells sense ECM changes through cell surface localised receptors, such as integrins. Integrins are heterodimeric receptors consisting of one α subunit, one β subunit [6,7]. In vertebrates, there are 18 α- and 8 β-subunits that give rise to 24 known integrins [8,9]. Both subunits consists of an intracellular and an extracellular domain, and a single-pass transmembrane domain [6]. Once activated, the extracellular domain can anchor cells to various types of ECM molecules, where the binding specificity depends on the α- and β-subunit combination [8]. The intracellular domain physically links to the cells’ cytoskeleton and simultaneously activates signalling events [8]. Thus, binding between integrins and ligands can trigger intracellular signalling, where integrins are known to mediate both inside-out and outside-in signalling. The inside-out signalling activates integrins from within the cells, extending the integrin into its active form [7]. Outside-in signalling is induced through integrin-binding to, e.g., ECM proteins, triggering signalling pathways including the FAK/SRC, PI3K/AKT and MAPK/ERK pathways via the cytoplasmic tail of the integrin [6,7]. This gives integrins the power to change both cell morphology, motility and gene expression simply through ECM binding. Integrins are expressed in a cell type- and tissue-specific manner, where expression is rapidly regulated with changes in the cellular environment [8]. Integrins can be categorised into four main groups according to their ligand-binding specificities: laminin-, collagen-, and RGD-binding integrins, in addition to leukocyte-specific integrins [7]. Integrin α8β1 belongs to the RGD-binding group of integrins which recognises proteins containing the tripeptide Arg (R)—Gly (G)–Asp (D), where the RGD-containing ECM protein nephronectin (NPNT) is believed to be the most prominent. NPNT not only provides a structural foundation for cells through its incorporation into the basement membrane, but also triggers intracellular signalling in a spatiotemporal manner that is particularly important during differentiation and development. Such processes are often exploited by cancer cells and they are therefore important to fully understand to enable therapeutic intervention. To understand the role of NPNT in cancer progression, we will start by reviewing the structure and domain-related functions of NPNT. Thereafter, we will discuss expression and regulation of the protein, and review studies that explore the roles of NPNT in vivo with special emphasis on cancer-related processes, and in particular metastasis.

## 2. Nephronectin (NPNT)

NPNT was originally identified as an ECM protein by two independent research groups in 2001 [10,11]. Brandenberger et al. reported NPNT to be involved in the embryonic development of the kidney, hence the name nephronectin (nephron: unit within the kidney; nectin: cellular adhesion proteins) [11]. Morimura and colleagues discovered the same protein to be associated with osteoblast differentiation, and named it preosteoblast epidermal growth factor-like repeat protein with meprin, A5 protein and receptor protein-tyrosine phosphatase µ domain (POEM) [10]. For this review, we will use the name nephronectin (NPNT).

## 3. NPNT Structure and Domain Related Functions

**Figure 1:**
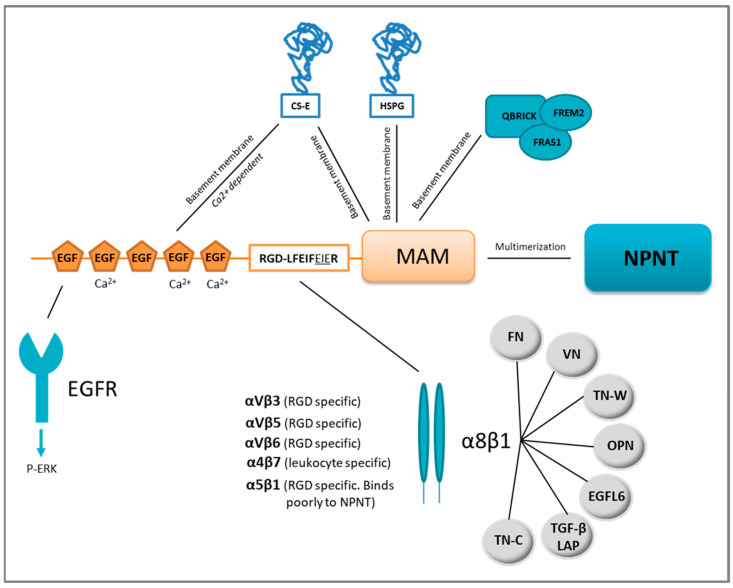
Nephronectin (NPNT) structure, domain-related binding partners and NPNT–integrin redundancy. NPNT consists of three main domains, the N-terminal EGF-like repeats, the linker-region containing two integrin-binding motifs, and the C-terminal MAM domain. The EGF-like repeats may bind the epidermal growth factor receptor (EGFR) and trigger intracellular signalling via ERK phosphorylation (P). Chondroitin sulphate-E (CS-E) can also bind to NPNT via the EGF-like repeats and the MAM domain. The MAM domain is responsible for binding to heparan sulphate proteoglycans (HSPG) in the basement membrane. NPNT may also bind QBRICK, FRAS1 and FREM2 in the basement membrane via its MAM domain. NPNT may multimerise via the MAM domain, and bind several different integrins, most prominently α8β1, which may furthermore associate with several other binding partners. FN = fibronectin, VN = vitronectin, OPN = osteopontin, TN-C = tenascin-C, TN-W = tenascin-W, TGF-β LAP= transforming growth factor-β latency-associated protein.

NPNT was first reported to be a secreted and glycosylated ECM protein of 70–90 kDa. It was later discovered that NPNT is also located intracellularly [12]. NPNT comprises three functional domains: the five N-terminal epidermal growth factor (EGF)-like repeats, a central linker region with a proline-rich, mucin-like region containing two integrin-binding sequences, and a C-terminal MAM domain (Figure 1) [11,13]. An NPNT homolog also exists, termed epidermal growth factor-like protein 6 (EGFL6, also known as MAEG). However, EGFL6 lacks one of the integrin-binding motifs (LFEIFEIER) [14,15]. The combination of these domains gives NPNT diverse functions which are summarised below.

### 3.1. EGF-Like Repeats

NPNT contains five EGF-like repeats [11], which are evolutionarily conserved protein domains found in many extracellular proteins [16] including tenascin-C [17], fibrillin-1 [18], jagged 1 [19] and factor IX [20]. Three of the EGF-like repeats of NPNT are believed to be Ca^2+^-binding [21], a property that is usually associated with interactions of similar domain types, but may also involve other structural motifs [16]. Some EGF-like repeats can bind and activate the EGF receptor (EGFR), triggering proliferation, migration and differentiation [17]. The EGF-like repeats of NPNT were assumed to bind the EGFR, as they were reported to initiate PI3K-AKT signalling downstream of EGFR in dental epithelial stem cells [22]. The EGF-like repeats of NPNT also induce differentiation of pre-osteoblastic cells into mature osteoblasts through ERK phosphorylation [23,24]. More recently, the EGF-like repeats of NPNT were shown to induce angiogenesis in vitro through ERK and p38 MAPK phosphorylation [25]. The NPNT homolog EGFL6 is also reported to induce angiogenesis [26]. Another reported binding partner of the NPNT EGF-like repeats is the ECM protein chondroitin sulphate-E (CS-E) (Figure 1), a binding partially dependent on Ca^2+^, as it can be reduced by the addition of EDTA. It has been hypothesised that the binding of NPNT to CS-E is needed for deposition of NPNT into basement membranes [27].

### 3.2. Linker Region (RGD- and LFEIFEIER Peptide Sequences)

Mucins are a family of large and highly O-glycosylated proteins, prominent in the glycocalyx of mucosal epithelia where they are located at the epithelial cell surface. Mucins are important for protective barrier functions of the mucosa and expressed in tissues like the intestine, respiratory tract, eye and middle ear epithelium [28]. The linker region of NPNT resembles a mucin, rich in proline, serine and threonine and highly O-glycosylated with a sialic acid cap [11,13,29]. One N-glycosylation site is also predicted to be present in this mucin-like region [11]. The N-terminal two-thirds of the linker region harbours most of the glycosylations [13], while the remaining portion contains two integrin-binding motifs: RGD (Arg-Gly-Asp) and LFEIFEIER (Leu-Phe-Glu-Ile-Phe-Glu-Ile-Glu-Arg). Integrins reported to bind NPNT are integrin α5β1, αVβ3, αVβ5, αVβ6, α4β7 and α8β1 [11,30] as shown in Figure 1. Of these, the NPNT–α8β1 is best studied and believed to be most prominent, partly due to the overlapping phenotypes of the α8β1 and NPNT knock-out mice [31,32].

The RGD motif of NPNT is critical, however not efficient to secure the binding of NPNT to integrin α8β1 [13]. The LFEIFEIER-motif functions as an integrin-binding enhancer motif, which has thus far only been reported involved in enhancing the specific NPNT–α8β1 binding [13,30]. Sato et al. found that the minimal sequence needed to secure enhancer site binding was EIE (LFEIFEIER) [13], while Sanchez-Cortes et al., using a different method (“self-assembled peptides”), found that the two FEI (LFEIFEIER) motifs were the essential sites. Furthermore, Sanchez-Cortez and colleagues also found that both RGD and LFEIFEIER sites interred equally strong binding to the α8β1 integrin [30].

Using RGD blocking peptides and NPNT with mutated integrin-binding motifs, the RGD motif of NPNT was shown to facilitate cell attachment of cardiomyocytes, endothelial and breast cancer cells [12,33,34]. Furthermore, by mutating the integrin-binding motifs, metastasis of breast cancer cells was significantly reduced [12,34]. Through antibody blocking of the RGD and LFEIFEIER sequence, collagen-induced inflammatory arthritis was reduced [35]. The RGD and partially the LFEIFEIER sequence have also been suggested to be involved in recruitment of CD4+ T cells in both acute and chronic liver hepatitis [36].

### 3.3. MAM Domain

MAM domains are found in a variety of proteins, including ECM and several transmembrane proteins. The domain consists of approximately 170 amino acids and contains four cysteine residues that potentially form disulphide bridges [27,37]. MAM domains are assumed to contribute to the proteins’ adhesive properties [38], including cell–cell adhesion. When NPNT was expressed in fibroblast-like and pre-osteoblast cells, the protein was not released into the culture medium, but was assumed to be attached to the cell surface. By removing the MAM domain, the protein was released into the medium, indicating that the MAM domain was indeed involved in the cell surface localization of NPNT. When a mutated recombinant NPNT (RGD -> RGE), lacking the EGF domains, was used for coating in cell adhesion studies, leukemic-derived cells stably expressing integrin α8β1 showed reduced survival and cell spreading. Though the cells did not spread out, they adhered to the coated surface through binding to the remaining MAM domain. This shows that the MAM domain plays a role in the initial phases of cell adhesion [10]. Some MAM-containing proteins may form dimers through their MAM domains [39,40], where the NPNT MAM domain is likely involved in the formation of NPNT multimers [10,13]. The importance of multimeric NPNT is not known, but could be related to its localisation and functions in different basement membranes. Through the MAM domain, NPNT binds to basement membrane located proteins. These include heparan sulphate (HS) proteoglycans (HSPG), like agrin and perlecan, chondroitin sulphate (CS)-E, QBRICK, FRAS1 and FREM2 [27,41]. NPNT also binds the highly sulphated glycosaminoglycan, heparin [27]. The MAM domain was, however, not involved in binding to CS-A, CS-C, CS-D, dermatan sulphate or hyaluronan, see Figure 1 [27]. The importance of the localisation of NPNT in the basement membrane is exemplified in the study of Fraser syndrome. Fraser syndrome is a rare autosomal recessive multiorgan congenital disorder that leads to fused eyelids, fingers and toes as well as renal agenesis, pulmonary complications and diverse morphogenetic defects. When the basement membrane protein QBRICK is dysfunctional, it provokes Fraser syndrome. QBRICK facilitates the assembly of NPNT into the basement membrane (together with FRAS1 and FREM2), where the MAM domain ensures the binding. Without QBRICK or NPNT, integrin α8β1 binding to the basement membrane could not occur. Although the NPNT homolog EGFL6/MAEG was present, this could not secure the α8β1 binding. The authors showed that NPNT was a higher-affinity ligand for α8β1. Integrin α8β1 binding is important for cell differentiation and organ development and its loss leads to the widespread defects observed [32,41,42].

## 4. Expression and Functional Roles of NPNT

NPNT expression is restricted to specific sites during tissue development and tissue homeostasis, where it plays a pivotal role in cell differentiation [32,42,43]. NPNT is expressed in the developing mouse embryo in many different types of tissues. These include the developing renal tubules, parathyroid and thyroid glands, endocrine organs and choroid plexus of the brain, tooth germs, lens of the eyes, the basal lamina of the apical surface of the ear epithelia, Rathke’s pouch, basal lamina of the lip and skin epithelium, basal lamina of the lungs, stomach, developing bone, oesophagus and taste buds of the tongue. Expression is somewhat weaker in the muscles of the tongue, developing pancreas and the lobe of the liver [10,11]. NPNT knock-out mice (NPNT^−/−^) display renal agenesis and hypoplasia [11,32]. This phenotype resembled that of mice with non-functional integrin α8β1 [31], which triggered Reichardt and colleagues to test whether NPNT was the binding partner of integrin α8β1 [11,32]. Integrin α8β1 is a member of the RGD-binding group of integrins, where the α8 subunit associates exclusively with the β1 subunit [44,45]. The α8 subunit is expressed in vascular and visceral smooth muscle, liver stellate cells, and smooth muscle-like contractile cells. In addition, the kidney mesenchyme (mesangial cells) expresses α8, as well as one cell type in the alveolar wall of the lungs, most likely the alveolar myofibroblasts [44,45,46]. Müller et al. reported expression of the α8 subunit in mesenchymal but not epithelial cells of developing organs such as the gut, lung, gonads and the nephrogenic cord [31]. A crucial role for α8β1 has been uncovered in early steps of kidney morphogenesis in both mice [31] and humans [47]. Integrin α8^−/−^ knock-out mice display frequent renal agenesis or hypoplasia, but also inner ear hair cell (stereocilia) defects [7,31]. Linton et al. could show that the NPNT–α8β1 interaction plays an important role in embryonic kidney development, and is required for the invasion of the metanephric mesenchyme by the epithelial cells of the uretic bud during kidney development. The downstream signalling was shown to be elicited through the glial cell line-derived neurotrophic factor (GDNF), a member of the TGF-β superfamily [32]. Although NPNT is expressed in many specific tissues during development, only the kidneys show macroscopic abnormalities in NPNT^−/−^ mice. This indicates that there is a functional redundancy of the NPNT–integrin α8β1 interaction (Figure 1) [32]. In addition to NPNT, α8β1 can bind [10,11,13] fibronectin (FN) [48], vitronectin (VN) [46], tenascin-C (TN-C) [49], osteopontin (OPN) [50], tenascin-W (TN-W) [51] and the latency-associated peptide (LAP) of transforming growth factor-β1 (TGF-β1) [52] and EGFL6 [42]. Functional redundancy of the NPNT–integrin α8β1 interaction has been confirmed studying hair follicles of the epidermis [42]. Fujiwara et al. found that the stem cells in the hair follicle bulge deposited NPNT into the underlying basement membrane. Mesenchymal cells expressing integrin α8β1 adhered to NPNT and up-regulated smooth muscle markers, triggering arrector pili muscle cell transformation. However, in NPNT^−/−^ mice, the arrector pili muscle cells adhered, not to the bulge, but rather to the follicle above the bulge where the NPNT homolog, EGFL6, was expressed [42]. A similar functional redundancy was observed in the developing heart of zebrafish. NPNT is an upstream regulator of Bmp2-Has2 signalling, and inhibition of the signalling cascade caused failure of heart valve formation. However, integrin α8β1 was not found expressed in the tissue and was therefore most probably not involved in the process [53], indicating the involvement of an alternative receptor.

Whereas the function of NPNT in renal development is well established, much less is known about the function of NPNT expression in the choroid plexus of the brain, lens of the eye, parathyroid and thyroid glands, and the basal lamina of the apical surface of the ear epithelia. Interestingly, α8^−/−^ knock-out mice also display inner ear hair cell (stereocilia) defects [31]. This warrants further investigation. NPNT is also heavily expressed in lung tissue. Increased serum levels of NPNT are linked to silicosis, a type of lung fibrosis [54]. Furthermore, through genome-wide association meta-analysis, NPNT was one of several genes associated with lung function and the susceptibility of developing chronic obstructive pulmonary disease (COPD) [55,56]. A recent preprint indicates that a splice variant of NPNT includes an additional serine residue in the splice site, and is associated with COPD. The serine residue is predicted to be located between the signal peptide and the first EGF-like repeat [57]. The structure and function of this newly discovered variant of NPNT is still to be resolved. NPNT is also elevated in diabetic glomerulosclerosis and nephropathy [58,59] and a maker for glomerular regeneration [60].

As previously reviewed by Sun et al., expression of the NPNT gene is regulated by several different signalling pathways [61]. As summarised in Table 1, *NPNT* is induced and repressed by a diverse spectrum of factors.

Most studies on NPNT regulation (Table 1) are done on osteoblasts, where the protein has a central role in differentiation. However, different effects might be expected in cancer cells. This remains to be tested. Exogenously supplied NPNT induces osteoblast differentiation in pre-osteoblast cells, and the process is mediated through the EGF-like repeats [24]. Fang and colleagues showed that TGF-β1 inhibits NPNT-induced osteoblast differentiation [23]. TGF-β1 stimulates the early steps of osteoblast differentiation, but inhibits the final steps [76]. Cultured pre-osteoblasts stimulated with TGF-β1 down-regulated NPNT expression and differentiation was inhibited [64,77]. The suppression was transferred through TGF-β1-induced phosphorylation of ERK1/2 and JNK in the osteoblasts [64]. It has also been shown that microRNA (miR) plays a part in the regulation of NPNT and osteoblast differentiation [69,78]. Yang and colleagues found five potential miRNAs that can bind to the 3′UTR of *NPNT* and by that regulate osteoblast differentiation: these included mmu-miR23a, 101a, 296-5p, 328 and 425. The authors suggested that the regulation was through GSK3β, Cyclin D and ERK [69]. Wnt3a is one of the signalling molecules reported to up-regulate NPNT expression in the human pre-osteoblastic cell line MC3T3-E1. The up-regulation was mediated through β-catenin signalling [70], a finding supported by the coinciding observation that NPNT expression is up-regulated in the K14∆β-cateninER mice where a stabilised β-catenin is expressed [42].

## 5. The Metastasis-Promoting Protein NPNT

Cancer cells have the ability to exploit mechanisms that are normally used during embryogenesis and differentiation to increase the cancer cells’ ability to thrive and spread, of which the most well-known is epithelial-to-mesenchymal transition. As NPNT seems to play a pivotal role in organogenesis and differentiation, it is not surprising that the protein can be exploited also during cancer progression. Simple alterations in the ECM composition in cancer may very well impact on cancer cell survival, proliferation, migration, invasion and metastasis.

A role for NPNT has been suggested in several different cancer types, including promoting migration, invasion and poor prognosis in gastric cancer [79], promoting bone metastasis in breast cancer [80], and promoting anti-adhesive and anti-apoptotic behaviour to human adrenocortical carcinoma cells [71]. NPNT also induced hormone production (aldosterone) in a type of adenoma, indicating that NPNT has diverse functional roles also in cancer [71]. Low levels of NPNT in Laron syndrome was correlated to low prevalence of cancer in a large epidemiological study of individuals with this syndrome. Though insulin growth factor 1 is down-regulated in Laron syndrome and induces NPNT expression [74], a cancer-protective role of NPNT still needs verification. Though most studies link NPNT to cancer progression, one study shows that a down-regulation of NPNT in malignant melanoma was linked to cancer progression [81]. Most notably invasion and migration was reduced when NPNT was reintroduced into cancer cells, and cell adhesion was increased [81].

A role for NPNT in promoting breast cancer metastasis was first reported in 2005 by Eckhardt and colleagues [82]. Five breast cancer cell lines, each with a different propensity to metastasise, were injected into the mammary fat pad of syngeneic BALB/c mice. Distant metastasis was scored and the five cell lines were classified as either non-metastatic (67NR), weakly metastatic (168FARN and 66cl4) or highly metastatic cells (4T1.2 and 4T1.13). Analysis of differential gene expression showed that the highly metastatic cells had significantly higher expression of NPNT. When NPNT was knocked down using shRNA, the rate of metastasis was reduced, indicating that NPNT plays a role in promoting metastasis [82]. More recently, using formalin-fixed and paraffin-embedded tissues from a well-characterised cohort of 842 Norwegian women diagnosed with invasive breast cancer (1961–2008) [83], we showed that NPNT protein was expressed in more than 70% of all primary breast cancers. A granular intracellular staining pattern was correlated to poor prognosis. No expression of NPNT was found in normal breast tissue [12,34]. A similar granular staining pattern was found in mouse breast cancer tissues, and mouse breast cancer cells secreted small extracellular vesicles containing NPNT. Another intriguing finding was the selected packaging of a truncated form of NPNT (~20 kDa) into small extracellular vesicles secreted from mouse breast cancer cells. The truncated NPNT most likely corresponded to the MAM domain [29]. Additionally, Morimura et al. reported a similar truncated (~25–30 kDa) and secreted form of NPNT [10]. The truncated NPNT was selectively concentrated into the small extracellular vesicles [29], indicating that the truncated version could have a specific function. Certain small extracellular vesicles can create pre-metastatic niches, enabling circulating cancer cells to seed and colonise in initially inhabitable soils [84]. Whether these small extracellular vesicles containing NPNT create such a pre-metastatic niche needs to be tested. We did, however, find that mouse breast cancer cells overexpressing wild type NPNT metastasised more readily to the lungs compared to control cells. However, when the integrin-binding motifs were mutated (RGD -> RGE and EIE -> AIA), the ability to colonise the lungs was reduced. These results showed that the integrin-binding motifs play a direct role in promoting metastasis [12]. The cancer cells were tail vein injected, bypassing the first steps of metastasis (invasion and intravasation), hence we could only conclude on the role of NPNT in the final steps of metastasis (survival in the circulation, extravasation or colonization). More recently we reported that the integrin-binding motifs of NPNT play a role specifically in extravasation during breast cancer brain metastasis. NPNT increased the cells’ ability to bind to—and migrate through—brain endothelial cells. Though more work is needed to validate these findings, this indicates that NPNT may play a part in transmigration of the endothelium [34]. Hypothetically, NPNT could function as a bridging molecule between disseminating cancer cells and the endothelial wall. In support of this hypothesis, once secreted, NPNT may bind to its own cell surface either via the MAM domain or the integrin-binding motifs [10,34]. NPNT harbours five EGF-like repeats that may be able to bind EGFR [22]. Additionally, both the EGF-like domains and the MAM domain are known to bind basement membrane proteins such as HSPG, CS, QBRICK, FRAS1 and FREM2, where CS-E is known to be expressed in the brain, kidney, cartilage and hair follicles [27]. Taken together, NPNT may promote metastasis through several mechanisms, including increased survival, angiogenesis, migration, extravasation and colonization.

## 6. Future Perspectives

The interactions between the extracellular matrix and receptors such as integrins play important roles in several different types of diseases such as thrombosis, inflammation, fibrosis and cancer. These interactions pose as desired drug targets especially due to their accessibility [85]. However, many integrin-targeting drugs have failed in clinical trials. Cilengitide, targeting the RGD motif, failed due to lack of efficiency [86,87]. In this regard, the NPNT—Integrin α8β1 interaction may present an alternative target for therapy. NPNT induced viability of cancer cells by activating the p38 MAPK pathway through its integrin-binding motifs. Interestingly, it was mainly the LFEIFEIER-enhancer motif that caused the activation of p38 MAPK and increased cell viability in breast cancer cells [88]. Our results indicated that NPNT plays a role during metastasis and in particular extravasation [34], and by mutating the RGD site, some of the metastatic burden was reduced. However, when both integrin motifs were mutated, the metastasis was reduced to baseline levels [12]. This indicates a particular important role of the LFEIFEIER-motif in the metastatic process. Another issue when targeting integrins is redundancy. In the case of the LFEIFEIER-motif it is, to the best of our knowledge, only reported to enhance the NPNT–Integrin α8β1 binding. Although redundant interactions of integrin α8β1 exist, they may not always be “functional”. As shown by Kiyozumi et al., the NPNT homolog MAEG/EGFL6, which lacks the LFEIFEIER-motif, was unable to secure the α8β1 binding even though it contains the common RGD-motif [41]. We therefore propose that the LFEIFEIER-motif could be an attractive therapeutic target in cancers with high levels of NPNT. Due to the additional importance of the RGD motif, a combinatorial treatment along with inhibitors such as cilengitide might be an attractive approach. To the best of our knowledge, such inhibitors do not exist and warrants further investigation.

Thus far, NPNT is reported to play a role in a limited number of cancer types, but given its many domain-related functions and specific expression pattern, we believe many more important roles are still to be uncovered.

## Figures and Tables

**Table 1:** Expression and repression of the NPNT gene. Regulation is indicated by colour-coding: up = green, down = red. The downstream signalling pathways/effector(s) are shown and their references. ERK = extracellular signal-regulated kinase, MAPK = mitogen-activated protein kinase, ALK = anaplastic lymphoma kinase, JNK = c-Jun N-terminal kinase, PI3K = phosphoinositide 3-kinase, Klf2 = Kruppel-like factor 2, FGFR = fibroblast growth factor, JAK = Janus kinase, STAT = signal transducer and activator of transcription proteins, TCF = T-cell specific transcription factor, LEF = lymphoid enhancer binding factor, Wnt = wingless-related integration site, IGFR = insulin growth factor receptor, Sp1 = specificity protein 1.

Regulatory Effect on NPNT	Initiator(s)	Downstream Effector(s)	References
Down-regulation	Tumour necrosis factor α (TNF α )	Nuclear factor-κB (NF-κB)	[62]
	Transforming growth factor β (TGF- β )	ALK5, Smad2 ERK1/2, JNK, MAPK	[23,63,64]
	Fibroblast growth factor-2 (FGF-2)	JNK PI3K	[65]
	Interleukin-1 β (IL-1 β )	JNK ERK1/2	[66]
	Oncostatin M (OSM)	JAK/STAT MAPK	[67]
	Inorganic Phosphate	FGFRs (via Pit)	[68]
	Mmu-miRNAs 23a, 101a, 296-5p, 328, 425	GSK3β, Cyclin D, ERK	[69]
Up-regulation	Wnt3a Wnt	β-catenin, TCF/LEF	[42,70,71]
	Bone morphogenetic protein-2 (BMP-2)		[72]
	Vitamin D_3_	Vitamin D receptor (VDR)	[73]
	Fibroblast growth factor 10 (FGF10)	transcription factor T box 5 (Tbx5)	[43]
	Insulin growth factor (IGF)	IGFR—ERK1/2	[74]
	?	ERK5—Sp1—Klf2/4	[75]
